# Significance of intrinsic breast cancer subtypes on the long-term prognosis after neoadjuvant chemotherapy

**DOI:** 10.1186/s12967-018-1679-0

**Published:** 2018-11-09

**Authors:** Wataru Goto, Shinichiro Kashiwagi, Koji Takada, Yuka Asano, Katsuyuki Takahashi, Hisakazu Fujita, Tsutomu Takashima, Shuhei Tomita, Kosei Hirakawa, Masaichi Ohira

**Affiliations:** 1 Department of Surgical Oncology, Osaka City University Graduate School of Medicine, 1-4-3 Asahi-machi, Abeno-ku, Osaka, 545-8585 Japan; 2 Department of Pharmacology, Osaka City University Graduate School of Medicine, 1-4-3 Asahi-machi, Abeno-ku, Osaka, 545-8585 Japan; 3 Department of Scientific and Linguistic Fundamentals of Nursing, Osaka City University Graduate School of Nursing, 1-5-17 Asahi-machi, Abeno-ku, Osaka, 545-0051 Japan

**Keywords:** Breast cancer, Neoadjuvant chemotherapy, Post-recurrence survival, Intrinsic subtype, Long-term prognosis

## Abstract

**Background:**

The prognosis of breast cancer and the treatment response to neoadjuvant chemotherapy (NAC) differ depending on the intrinsic molecular subtypes. We evaluated the prognostic significance of immunohistological subtypes in patients with recurrent breast cancer after treatment with NAC and surgery.

**Methods:**

A total of 237 patients with breast cancer treated with NAC and subsequent curative surgery between 2007 and 2015 were analyzed. The correlation between intrinsic molecular subtypes and clinicopathological features, prognosis, and pathological complete response (pCR) rate of NAC were investigated retrospectively.

**Results:**

There were 55 (23.2%) patients with recurrence after surgery. No significant difference in post-recurrence survival (PRS) was noted among the subtypes (p = 0.397). In patients with estrogen receptor-positive human epidermal growth factor receptor (HER) 2-negative (luminal) malignancy, PRS was significantly better in the pCR group than in the non-pCR group (p = 0.031). Conversely, pCR was not a significant predictor of improved PRS in patients with triple-negative breast cancer (TNBC; p = 0.329). Multivariate analysis revealed that the efficacy of NAC [hazard ratio (HR) 300.204, p < 0.001] and the initial metastasis site (HR 15.037, p = 0.005) were independent predictors for PRS in patients with luminal breast cancer, while Ki-67 (HR 51.171, p = 0.020) and the initial metastasis site (HR 13.318, p = 0.048) were independent predictors for PRS in patients with TNBC.

**Conclusions:**

The prognostic factors for each intrinsic subtype should be evaluated separately in patients with recurrent breast cancer following NAC and surgery.

## Background

Breast cancer is a heterogeneous disease. Based on gene expression profiling derived from complementary deoxyribonucleic acid (cDNA) microarrays, breast cancer is classified into distinct molecular subtypes, and this diversity is clinically useful in obtaining prognostic information [1].

Treatment with neoadjuvant chemotherapy (NAC) increases the rate of breast-conserving surgery and reduces the risk of postoperative recurrence in patients with resectable breast cancer [2, 3]. In particular, pathological complete response (pCR) after NAC is an independent predictor of favorable outcome in human epidermal growth factor receptor (HER) 2-enriched and triple-negative breast cancer (TNBC) subtypes [4–9].

Although recurrence and metastasis remain the major problems in curative treatment [10], some patients with recurrent breast cancer have a relatively good post-recurrence survival (PRS). PRS is primarily related to tumor biology, including intrinsic subtypes.

However, few studies have examined the relationship between intrinsic subtypes and the PRS of patients with recurrent breast cancer [11–14]. This study aimed to evaluate the clinicopathological features and PRS according to intrinsic molecular subtypes of breast cancer in patients treated with NAC and subsequent curative surgery. To our knowledge, this study is the first to demonstrate the prognostic significance of immunohistological subtypes in patients with recurrent breast cancer after treatment with NAC and surgery.

## Methods

### Patients

This study included 237 patients with resectable, early-stage breast cancer diagnosed as stage IIA (T1, N1, M0 or T2, N0, and M0), IIB (T2, N1, M0 or T3, N0, and M0), or IIIA (T1-2, N2, M0 or T3, N1-2, and M0) treated with NAC between 2007 and 2015. Tumor stage and T and N factors were stratified based on the TNM Classification of Malignant Tumors, UICC Seventh Edition [15]. Breast cancer was confirmed histologically via core needle biopsy, or ultrasonography-guided vacuum-assisted biopsy. The tumor stage was determined via systemic imaging studies using computed tomography and bone scintigraphy. Tumors were classified into intrinsic breast cancer subtypes according to the immunohistochemical expression of estrogen receptor (ER), progesterone receptor (PgR), HER-2, and Ki-67. The cut-offs for ER and PgR positivity were both > 0% positive tumor cells with nuclear staining. Tumors with (1) 3+ HER2 on immunohistochemical staining, (2) HER2/centromere 17 ratio of ≥ 2.0 [16, 17], and a (3) a Ki-67 labeling index of ≥ 14% tumor cells on nuclear staining, were considered to exhibit HER2 overexpression [18]. Meanwhile, tumors with a 2+ HER2 on immunohistochemical staining were analyzed further via fluorescence in situ hybridization.

All patients received a standard NAC protocol consisting of four courses of FEC 100 (500 mg/m^2^ fluorouracil, 100 mg/m^2^ epirubicin, and 500 mg/m^2^ cyclophosphamide) every 3 weeks, followed by 12 courses of 80 mg/m^2^ paclitaxel administered weekly [19, 20]. Sixty-four patients had HER2-positive breast cancer and were administered additional [weekly (2 mg/kg) or tri-weekly (6 mg/kg)] trastuzumab during the paclitaxel treatment [21]. Chemotherapy was administered in the outpatient department. Therapeutic anti-tumor effects were assessed according to the Response Evaluation Criteria in Solid Tumors [22]. pCR was defined as the complete disappearance of the invasive compartment of the lesion with or without intraductal components, including the lymph nodes [2]. Patients underwent mastectomy or breast-conserving surgery after NAC. All patients who underwent breast-conserving surgery also underwent postoperative radiotherapy of the remnant breast. Relapse-free survival (RFS) was defined as the absence of all local, loco-regional, and distant recurrences. Conversely, PRS was defined as the time from tumor relapse to death from any cause. All patients were followed up via physical examination every 3 months, with ultrasonography every 6 months, and computed tomography and bone scintigraphy annually.

### Ethics statement

This study was conducted at Osaka City University Graduate School of Medicine, Osaka, Japan, according to the Reporting Recommendations for Tumor Marker Prognostic Studies guidelines and following a retrospectively written research, pathological evaluation, and statistical plan [23]. The study protocol was approved by the Ethics Committee of Osaka City University. Written informed consent was obtained from all subjects (#926).

### Statistical analyses

Statistical analyses was performed using the JMP13 software program (SAS Institute, Cary, NC, USA). The associations between intrinsic breast cancer subtypes and clinicopathological variables were evaluated using the χ^2^ test (or Fisher’s exact test when necessary). The Kaplan–Meier method was used to estimate RFS and PRS. The association between breast cancer subtypes and survival was analyzed via Kaplan–Meier plots and log-rank testing. The Cox proportional hazards model was used to compute univariate and multivariate hazards ratios (HR) for the study parameters with 95% confidence interval (CI). A p value of < 0.05 was considered significant.

## Results

### Analyses of all breast cancer patients

**Fig. 1:**
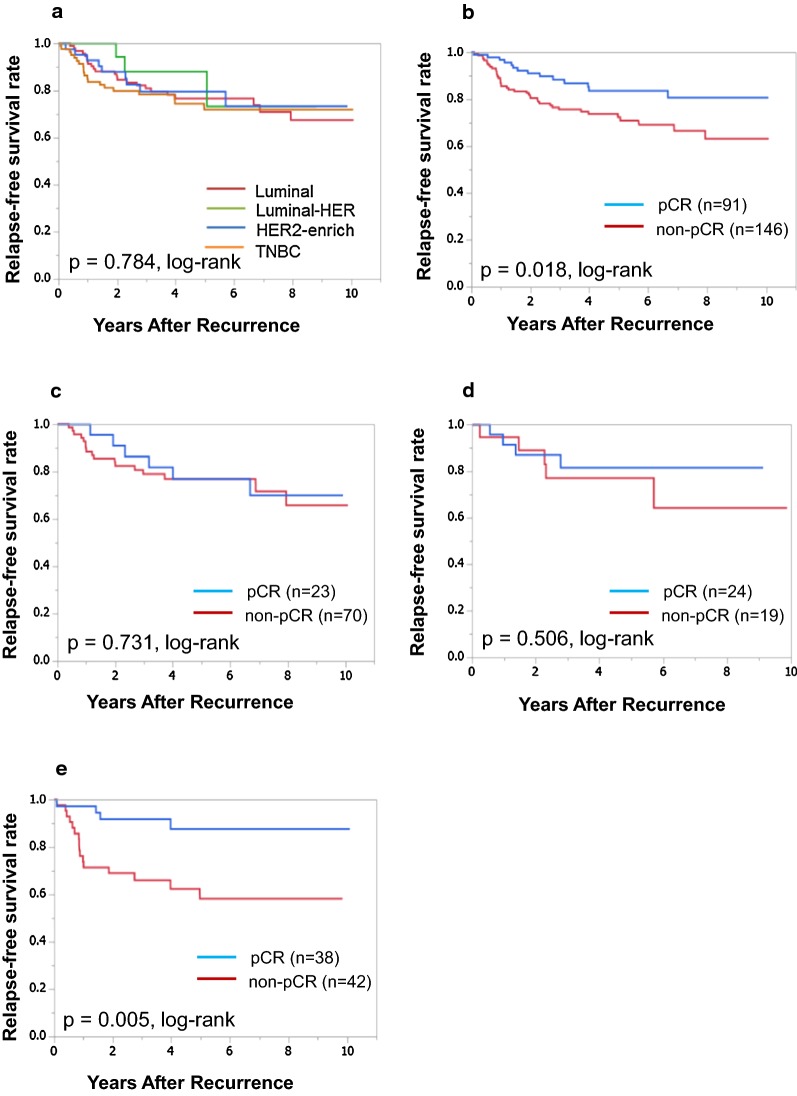
Relapse-free survival (RFS) for each intrinsic subtype and for pathological response. RFS was not significantly different in each subtype (p = 0.784, log-rank) (**a**). Patients who achieved pCR had significantly better RFS among all breast cancers (p = 0.018, log-rank) (**b**). RFS was not significantly differ between patients with pCR and non-pCR of Luminal breast cancer (p = 0.731, log-rank) (**c**). of HER2-enriched breast cancer (p = 0.506, log-rank) (**d**). Patients who achieved pCR had significantly better RFS of TNBC (p = 0.005, log-rank) (**e**)

The correlation between clinicopathological features and each intrinsic subtype is presented in Table 1. A total of 237 patients were included in this study. Among these, 93 (39.2%), 21 (8.9%), 43 (18.1%), and 80 (33.8%) had estrogen receptor-positive HER2-negative (luminal), luminal-HER2, HER2-enriched, and TNBC, respectively. Evaluation based on clinicopathological features showed that the pCR rate was significantly higher in patients with HER2-enriched breast cancer and TNBC (p = 0.001). The median follow-up period for RFS was 4.2 years (range 0.1–10.0 years). RFS was not significantly different in each subtype (p = 0.784, log-rank) (Fig. 1a), and it was also significantly longer in patients who achieved pCR than those who did not (p = 0.018, log-rank) (Fig. 1b). In univariate analysis, RFS exhibited a significant relationship with Ki-67 (HR 0.553, 95% CI 0.325–0.945, p = 0.031) and pathological response (HR 2.046, 95% CI 1.143–3.891, p = 0.015). Multivariate analysis revealed that Ki-67 (HR 0.548, 95% CI 0.300–0.991, p = 0.047) and pathological response (HR 1.886, 95% CI: 1.005–3.803, p = 0.048) were independent prognostic factors for recurrence (Table 2).

**Table 1:** Correlation between clinicopathological features and each intrinsic subtype in 237 patients treated with NAC

Parameters	Intrinsic subtype				p-value
	Luminal (n = 93)	Luminal-HER2 (n = 21)	HER2-enriched (n = 43)	TNBC (n = 80)	
Age at operation					
≤ 56	48 (51.6%)	12 (57.1%)	18 (41.9%)	39 (48.8%)	
> 56	45 (48.4%)	9 (42.9%)	25 (58.1%)	41 (51.2%)	0.641
Menopause					
Pre-	42 (45.1%)	9 (42.9%)	15 (34.9%)	29 (36.3%)	
Post-	51 (54.9%)	12 (57.1%)	28 (65.1%)	51 (63.7%)	0.564
Tumor size (cm)					
≤ 2	13 (14.0%)	5 (23.8%)	5 (11.6%)	9 (11.3%)	
> 2	80 (86.0%)	16 (76.2%)	38 (88.4%)	71 (88.7%)	0.546
Lymph node status					
Negative	20 (21.5%)	9 (42.9%)	14 (32.6%)	22 (27.5%)	
Positive	73 (78.5%)	12 (57.1%)	29 (67.4%)	58 (72.5%)	0.206
Nuclear grade					
1, 2	76 (81.7%)	18 (85.7%)	35 (81.4%)	65 (81.3%)	
3	17 (18.3%)	3 (14.3%)	8 (18.6%)	15 (18.7%)	0.969
Ki67 (%)					
≤ 14	34 (36.6%)	11 (52.4%)	15 (34.9%)	19 (23.8%)	
> 14	59 (63.4%)	10 (47.6%)	28 (65.1%)	61 (76.2%)	0.066
Pathological response					
Non-pCR	70 (73.8%)	15 (71.4%)	19 (44.2%)	42 (52.5%)	
pCR	23 (26.2%)	6 (28.6%)	24 (55.8%)	38 (47.5%)	0.001

*NAC* neoadjuvant chemotherapy, *HER2* human epidermal growth factor receptor 2, *TNBC* triple-negative breast cancer, *pCR* pathological complete response

**Table 2:** Univariate and multivariate analyses with respect to relapse-free survival in breast cancer subtypes

	Univariate analysis			Multivariate analysis		
	Hazard ratio	95% CI	p-value	Hazard ratio	95% CI	p-value
All breast cancer (n = 237)						
Age (≤ 56)	1.491	0.877–2.565	0.141	2.130	0.828–4.847	0.111
Menopause (−)	1.220	0.709–2.074	0.467	0.703	0.310–1.802	0.439
Tumor size (> 2)	1.679	0.738–4.830	0.236	1.509	0.640–4.436	0.372
Lymph node (+)	1.720	0.904–3.620	0.102	1.829	0.950–3.880	0.072
Nuclear grade (3)	0.899	0.427–1.713	0.758	1.452	0.642–3.064	0.356
Ki67 (> 14)	0.553	0.325–0.945	0.031	0.548	0.300–0.991	0.047
Pathological response (non-pCR)	2.046	1.143–3.891	0.015	1.886	1.005–3.746	0.048
Luminal (n = 93)						
Age (≤ 56)	1.223	0.537–2.871	0.631	5.007	0.988–20.715	0.052
Menopause (−)	0.864	0.367–1.974	0.730	0.268	0.072–1.290	0.094
Tumor size (> 2)	2.077	0.607–13.00	0.276	2.444	0.650–16.093	0.206
Lymph node (+)	3.188	0.935–19.94	0.066	4.842	1.336–31.230	0.013
Nuclear grade (3)	1.110	0.365–2.799	0.839	2.386	0.640–8.278	0.187
Ki67 (> 14)	0.587	0.256–1.360	0.209	0.336	0.119–0.906	0.031
Pathological response (non-pCR)	1.178	0.487–3.281	0.728	1.403	0.513–4.387	0.523
HER2-enriched (n = 43)						
Age (≤ 56)	2.124	0.559–8.614	0.262	1.059	0.050–9.029	0.962
Menopause (−)	1.877	0.463–7.126	0.359	6.241	0.451–175.683	0.177
Tumor size (> 2)	1.063	0.195–19.750	0.953	0.281	0.024–6.713	0.368
Lymph node (+)	1.859	0.447–12.523	0.417	1.683	0.292–15.232	0.579
Nuclear grade (3)	0.405	0.022–2.245	0.344	0.163	0.007–1.387	0.103
Ki67 (> 14)	0.607	0.160–2.460	0.464	0.868	0.140–6.349	0.882
Pathological response (non-pCR)	1.556	0.412–6.289	0.508	4.430	0.569–55.264	0.163
TNBC (n = 80)						
Age (≤ 56)	1.483	0.611–3.697	0.381	2.697	0.542–10.890	0.205
Menopause (−)	1.318	0.515–3.195	0.550	0.539	0.136–2.660	0.416
Tumor size (> 2)	1.155	0.333–7.263	0.844	0.643	0.160–4.296	0.596
Lymph node (+)	1.065	0.411–3.282	0.904	0.625	0.214–2.083	0.421
Nuclear grade (3)	1.098	0.314–2.999	0.868	2.909	0.679–11.523	0.143
Ki67 (> 14)	0.488	0.202–1.250	0.130	0.486	0.150–1.518	0.213
Pathological response (non-pCR)	4.251	1.557–14.857	0.004	5.013	1.612–19.386	0.004

Values in parentheses are 95% confidence intervals

*CI* confidence interval, *pCR* pathological complete response, *TNBC* triple-negative breast cancer

Additionally, we investigated the prognostic factors for RFS in each breast cancer subtype. Among the 93 patients with luminal type, no significant difference was observed in RFS according to pathological response (p = 0.731, log-rank) (Fig. 1c). In univariate analysis, no clinicopathological feature correlated significantly with RFS. Meanwhile, multivariate analysis revealed that lymph node (HR 4.842, 95% CI 1.336–31.230, p = 0.013) and Ki-67 (HR 0.336, 95% CI 0.119–0.906, p = 0.031) were independent prognostic factors for recurrence (Table 2). Among the 43 patients with HER2-enriched breast cancer, no significant difference was noted in RFS in relation to pathological response (p = 0.506, log-rank) (Fig. 1d). In univariate and multivariate analyses, there was no independent prognostic factor for recurrence in this study (Table 2). Among the 80 patients with TNBC, RFS was significantly longer in patients who achieved pCR than those who did not (p = 0.005, log-rank) (Fig. 1e). In univariate analysis, only pathological response (HR 4.251, 95% CI 1.557–14.857, p = 0.004) was significantly correlated with RFS. Multivariate analysis also revealed that only pathological response (HR 5.013, 95% CI 1.612–19.386, p = 0.004) was an independent prognostic factor for survival. Because the number of patients with luminal-HER2 breast cancer was small (n = 21), statistical analyses were not performed (Table 2).

### Analyses of patients with recurrence after surgery

**Fig. 2:**
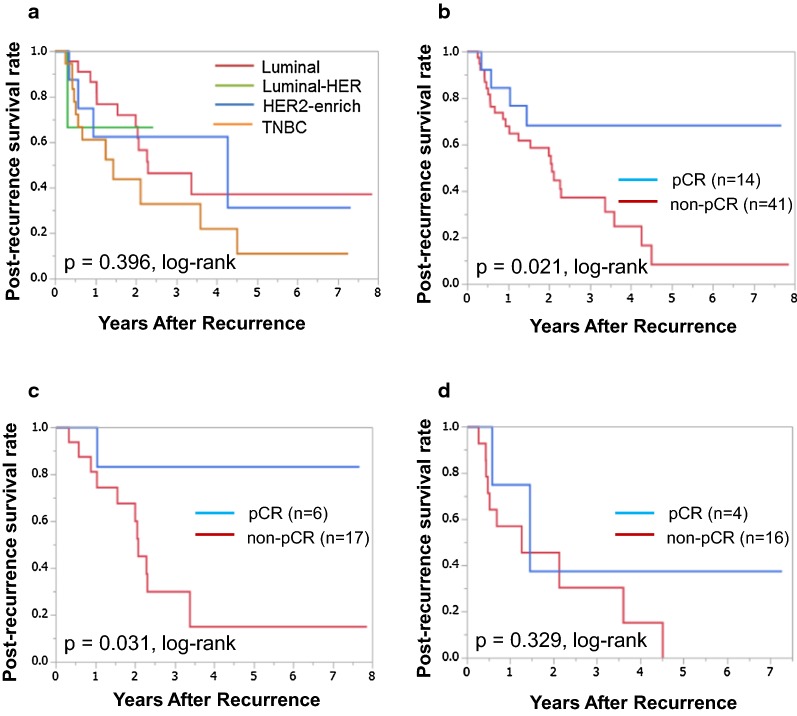
Post-recurrence survival (PRS) for each intrinsic subtype and for pathological response. Although TNBC was associated with the worst prognosis, PRS was not significantly different in each subtype (p = 0.396, log-rank) (**a**). Patients who achieved pCR had significantly better PRS among all breast cancers (p = 0.021, log-rank) (**b**). Patients who achieved pCR had significantly better PRS of Luminal breast cancer (p = 0.031, log-rank) (**c**). PRS was not significantly differ between patients with pCR and non-pCR of TNBC (p = 0.329, log-rank) (**d**)

Among the 237 patients, 55 relapsed after surgery. The correlation between clinicopathological features and each intrinsic subtype is presented in Table 3. Among the 55 patients who relapsed, 23 (41.8%), 3 (5.4%), 9 (16.4%), and 20 (36.4%) had luminal, luminal-HER2, HER2-enriched, and TNBC, respectively. Evaluation based on clinicopathological features revealed that there was no significant correlation between each intrinsic subtype and any clinicopathological parameter, including pCR (p = 0.306). The median follow-up period for PRS was 1.5 years (range 0.1–7.9 years). Although PRS was the worst in patients with TNBC, it was not significantly different in each subtype (p = 0.397, log-rank) (Fig. 2a). PRS was also significantly longer in patients who achieved pCR than those who did not (p = 0.021, log-rank) (Fig. 2b). In univariate analysis, PRS exhibited a significant relationship with pathological response (HR 3.321, 95% CI 1.264–11.437, p = 0.013) and RFS (HR 2.439, 95% CI 1.005–7.269, p = 0.049). Multivariate analysis showed that tumor size (HR 5.533, 95% CI 1.251–39.287, p = 0.023), Ki-67 (HR 2.606, 95% CI 1.028–6.780, p = 0.044), pathological response (HR 4.355, 95% CI 1.438–16.871, p = 0.008), and metastatic site (HR 2.496, 95% CI 1.007–6.126, p = 0.048) had strong prognostic significance for PRS (Table 4).

**Table 3:** Correlation between clinicopathological features and each intrinsic subtype in 55 patients with recurrence after surgery

Parameters	Intrinsic subtype				p-value
	Luminal (n = 23)	Luminal-HER2 (n = 3)	HER2-enriched (n = 9)	TNBC (n = 20)	
Age at operation					
≤ 56	13 (56.5%)	2 (66.7%)	5 (55.6%)	11 (55.0%)	
> 56	10 (43.5%)	1 (33.3%)	4 (44.4%)	9 (45.0%)	0.985
Menopause					
Pre-	10 (43.5%)	2 (66.7%)	4 (44.4%)	8 (40.0%)	
Post-	13 (56.5%)	1 (33.3%)	5 (55.6%)	12 (60.0%)	0.860
Tumor size (cm)					
≤ 2	2 (8.7%)	0 (0.0%)	1 (11.1%)	2 (10.0%)	
> 2	21 (91.3%)	3 (100.0%)	8 (88.9%)	18 (90.0%)	0.888
Lymph node status					
Negative	2 (8.7%)	1 (33.3%)	2 (22.2%)	5 (25.0%)	
Positive	21 (91.3%)	2 (66.7%)	7 (77.8%)	15 (75.0%)	0.437
Nuclear grade					
1, 2	18 (78.3%)	3 (100.0%)	8 (88.9%)	16 (80.0%)	
3	5 (21.7%)	0 (0.0%)	1 (11.1%)	4 (20.0%)	0.620
Ki67 (%)					
≤ 14	11 (47.8%)	3 (100.0%)	4 (44.4%)	8 (40.0%)	
> 14	12 (52.2%)	0 (0.0%)	5 (55.6%)	12 (60.0%)	0.175
Pathological response					
Non-pCR	17 (73.9%)	3 (100.0%)	5 (55.6%)	16 (80.0%)	
pCR	6 (26.1%)	0 (0.0%)	4 (44.4%)	4 (20.0%)	0.306
Metastatic site					
Local/bone	16 (69.6%)	2 (66.7%)	5 (55.6%)	12 (60.0%)	
Distant	7 (30.4%)	1 (33.3%)	4 (44.4%)	8 (40.0%)	0.866
Relapse-free survival (years)					
< 2	13 (56.5%)	1 (33.3%)	5 (55.6%)	16 (80.0%)	
≥ 2	10 (43.5%)	2 (66.7%)	4 (44.4%)	4 (20.0%)	0.220

*HER2* human epidermal growth factor receptor 2, *TNBC* triple-negative breast cancer, *pCR* pathological complete response

**Table 4:** Univariate and multivariate analyses with respect to post-recurrence survival in breast cancer subtypes

	Univariate analysis			Multivariate analysis		
	Hazard ratio	95% CI	p-value	Hazard ratio	95% CI	p-value
All breast cancer (n = 55)						
Age (≤ 56)	0.639	0.301–1.331	0.231	0.369	0.076–1.337	0.135
Menopause (−)	0.651	0.289–1.380	0.268	1.936	0.518–9.476	0.340
Tumor size (> 2)	2.262	0.666–14.138	0.216	5.534	1.251–39.387	0.023
Lymph node (+)	0.760	0.288–2.611	0.627	0.295	0.083–1.207	0.086
Nuclear grade (3)	1.308	0.516–2.921	0.547	0.751	0.256–2.016	0.577
Ki67 (> 14)	1.797	0.859–3.881	0.120	2.606	1.028–6.780	0.044
Pathological response (non-pCR)	3.321	1.264–11.437	0.013	4.355	1.438–16.871	0.008
Metastasis site (distant)	1.415	0.666–2.950	0.359	2.496	1.007–6.126	0.048
Relapse-free survival (< 2 years)	2.439	1.005–7.269	0.049	1.938	0.681–6.598	0.225
Luminal (n = 23)						
Age (≤ 56)	1.228	0.382–3.948	0.724	5.415	0.194–115.713	0.275
Menopause (−)	1.252	0.369–3.941	0.704	1.614	0.104–46.371	0.736
Tumor size (> 2)	6.235 × 10^7^	0.822–0.822	0.073	3.108 × 10^9^	1.662–7.5 × 10^179^	0.024
Lymph node (+)	5.733 × 10^7^	0.561–0.561	0.135	1.772 × 10^10^	1.806–unparsable	0.028
Nuclear grade (3)	1.198	0.265–4.042	0.791	0.436	0.034–4.914	0.477
Ki67 (> 14)	1.118	0.345–3.648	0.849	0.982	0.180–4.386	0.982
Pathological response (non-pCR)	7.144	1.358–131.592	0.016	300.204	7.824–52,372.311	< 0.001
Metastatic site (distant)	2.520	0.740–7.971	0.132	15.037	2.182–159.623	0.005
Relapse-free survival (< 2 years)	2.175	0.647–9.825	0.219	0.165	0.012–2.177	0.160
TNBC (n = 20)						
Age (≤ 56)	0.936	0.291–3.018	0.910	0.578	0.011–20.101	0.759
Menopause (−)	1.025	0.299–3.258	0.967	0.919	0.022–36.398	0.962
Tumor size (> 2)	0.948	0.222–6.663	0.949	2.141	0.100–93.753	0.639
Lymph node (+)	0.507	0.133–2.415	0.360	1.083	0.049–53.468	0.963
Nuclear grade (3)	1.841	0.479–6.155	0.350	0.109	0.003–1.421	0.094
Ki67 (> 14)	4.242	1.078–28.056	0.038	51.171	1.769–4346.194	0.020
Pathological response (non-pCR)	2.110	0.547–13.871	0.303	1.813	0.045–56.645	0.733
Metastatic site (distant)	1.569	0.486–5.070	0.442	13.318	1.021–540.473	0.048
Relapse-free survival (< 2 years)	7.070 × 10^7^	0.812–0.812	0.074	2.294 × 10^7^	0.027–3.0 × 10^289^	0.454

Values in parentheses are 95% confidence intervals

*CI* confidence interval, *pCR* pathological complete response, *TNBC* triple-negative breast cancer

Among the 23 patients with luminal type, PRS was significantly longer in patients who achieved pCR than those who did not (p = 0.031, log-rank) (Fig. 2c). Pathological response (HR 7.144, 95% CI 1.358–131.592, p = 0.016) was significantly correlated with PRS in univariate analysis. Multivariate analysis revealed that tumor size (HR 3.108 × 10^9^, 95% CI 1.662–7.5 × 10^179^, p = 0.024), lymph node (HR 1.772 × 10^10^, 95% CI 1.806–unparsable, p = 0.028), pathological response (HR 300.204, 95% CI 7.824–52,372.311, p < 0.001), and metastatic site (HR 15.037, 95% CI 2.182–159.623, p = 0.005) were independent prognostic factors for survival (Table 4). Among the 20 patients with TNBC, there was no significant difference in PRS in relation to pathological response (p = 0.329, log-rank) (Fig. 2d). In univariate analysis, only Ki-67 (HR 4.242, 95% CI 1.078–28.056, p = 0.038) was significantly correlated with PRS. Multivariate analysis revealed that Ki-67 (HR 51.171, 95% CI 1.769–4346.194, p = 0.020) and metastatic site (HR 13.318, 95% CI 1.021–540.473, p = 0.048) were independent prognostic factors for survival. Because the number of patients with luminal-HER2 (n = 3) and HER2-enriched (n = 9) breast cancer was small, statistical analyses were not performed (Table 4).

## Discussion

Classification of breast cancer intrinsic subtypes is useful in the prediction of therapeutic response and prognosis, mainly for the primary tumor. However, there have been few studies that stratify patients with recurrent breast cancer by intrinsic subtype [11–14]. These studies reported that ER-negative breast cancer or TNBC was significantly associated with poor prognosis. However, these investigations were not examined by each intrinsic subtype of breast cancer. In our study, among 20 TNBC patients with recurrence, low Ki-67 and metastatic site (local or bone) were significantly associated with good prognosis. In addition, although these studies included patients who were not treated with NAC, NAC is the gold standard of care for breast cancer. Our study is the first to investigate the prognostic factor of recurrent breast cancer after treatment with NAC and surgery in all intrinsic subtypes.

In the present study, patients with HER2-enriched breast cancer and TNBC had significantly higher pCR rates among all the patient groups. In particular, RFS after NAC and surgery was significantly longer for patients with TNBC who achieved pCR. Because some previous studies suggested that the treatment response of highly malignant breast cancers, namely, HER2-enriched breast cancer and TNBC, is related to the number of tumor-infiltrating lymphocytes, both are considered to have high immunoactivity [24–27]. However, among patients who relapsed after surgery, no association between each subtype and clinicopathological features was found, including pathological response. Additionally, PRS was significantly better for patients with luminal breast cancer who achieved pCR, but not for those with TNBC. Luminal breast cancer comprises epithelial cells and has low invasion capability, whereas TNBC comprises mesenchymal cells, and has high invasion and migration capabilities [28, 29]. Compared to that of the primary tumor, relapsed breast cancer after NAC and surgery often acquires resistance to chemotherapy; thus, the prognosis may depend on the degree of invasion or migration rather than immunoactivity.

In terms of metastatic sites at relapse, distant metastasis, such as the liver or lung, excluding bone, is considered life-threatening and is often treated with chemotherapy based on the first-line regimen [30]. Even in our study, the metastatic site was an independent prognostic factor for both the luminal and TNBC subtypes. A recent study reported that disease-free interval was significantly associated with PRS, particularly in luminal breast cancer [31]. However, in this study, there was no relationship between RFS and PRS for both luminal breast cancer and TNBC. The reason for this inconsistent result may be that we only analyzed patients treated with NAC. Future studies may find that cyclin-dependent kinase 4/6 inhibitors (palbociclib, ribociclib, and abemaciclib) could be an effective treatment modality for cases of recurrent luminal cancer that did not achieve pCR.

In the present study, patients were classified into subtypes according to findings from core needle biopsy before NAC, while it has been reported that the receptor status of the relapsed breast cancer after NAC may change [32, 33]. Re-biopsy after recurrence is recommended because it may improve information for creating an individualized treatment plan [16]. However, re-biopsy of distant recurrence is often difficult, including the brain or bone, and we did not analyze it in this study. Instead of core needle biopsy of the tumor, liquid biopsy, such as of circulating tumor cells, cell-free DNA, and tumor-derived exosomes, is likely to influence the treatment strategy in the future [34, 35].

A potential limitation of the current study is that we did not evaluate luminal-HER2, and HER2-enriched breast cancer in detail because of the small sample size of our study. Further prospective cohort studies are therefore needed to address these limitations.

## Conclusions

This study is the first to investigate the PRS of patients treated with NAC and subsequent curative surgery in each intrinsic molecular subtype. It is important to separately evaluate the prognostic factors for each intrinsic subtype in patients with recurrent breast cancer following NAC and surgery.

## Abbreviations

NAC: neoadjuvant chemotherapy
pCR: pathological complete response
PRS: post-recurrence survival
HER: human epidermal growth factor receptor
TNBC: triple-negative breast cancer
HR: hazard ratio
ER: estrogen receptor
PgR: progesterone receptor
RFS: relapse-free survival
CI: confidence interval
